# Scaling left ventricular mass in adolescent female soccer players

**DOI:** 10.1186/s12887-020-02043-7

**Published:** 2020-04-13

**Authors:** Diogo V. Martinho, João Valente-dos-Santos, Manuel J. Coelho-e-Silva, Arturo O. Gutiérrez, João P. Duarte, Pedro Lourenço-Farinha, Leonardo G. O. Luz, João Gonçalves-Santos, Dalmo R. L. Machado, Neiva Leite, Jorge Conde, Joaquim M. Castanheira, Sean P. Cumming, Lauren B. Sherar, Robert M. Malina

**Affiliations:** 1 Faculty of Sport Sciences and Physical Education, University of Coimbra, Coimbra, Portugal; 2 CIDAF (uid/dtp/04213/2020), University of Coimbra, Estadio Universitario, Pavilhao III, Coimbra, Portugal; 3 Faculty of Physical Education and Sport, Lusófona University, Lisbon, Portugal; 4 Sonora Institute of Technology, Sonora, Mexico; 5 LACAPS, Federal University of Alagoas, Arapiraca, Brazil; 6 Sports Medicine Centre, Portuguese Institute of Sports and Youth, Porto, Portugal; 7 School of Physical Education and Sport of Ribeirão Preto, University of São Paulo, Ribeirao Preto, Brazil; 8 Physical Education Department, Research Nucleus of Quality of Life, Federal University of Parana, Curitiba, Parana Brazil; 9 Department of Clinical Physiology, School of Health and Technology, Polytechnic Institute of Coimbra, Coimbra, Portugal; 10 Department for Health, University of Bath, Bath, UK; 11 School of Sport, Exercise and Health Sciences, Loughborough University, Loughborough, UK; 12 Department of Kinesiology and Health Education, University of Texas, Austin, USA; 13 School of Public Health and Information Sciences, University of Louisville, Louisville, KY USA

**Keywords:** Youth sport, Female athletes, Growth, Cardiac function, Skeletal age, Scaling

## Abstract

**Background:**

The aim of the study was to examine the contribution of chronological age (CA), skeletal maturation, training experience and concurrent body size descriptors, to inter-individual variance in left ventricular mass (LVM) among female adolescent soccer players.

**Methods:**

The sample included 228 female soccer players 11.8–17.1 years. Training experience defined as years of participation in competitive soccer (range 2–9 years), was obtained by interview. Stature, body mass and skinfolds (triceps, medial calf) were measured. Fat mass was estimated; Fat-free mass was derived. LVM was assessed by echocardiography. Skeletal maturity status was as the difference of skeletal age (SA, Fels method) minus CA.

**Results:**

Fat-free mass was the most prominent single predictor of LVM (R^2^ = 36.6%). It was associated with an allometric coefficient close to linearity (*k* = 0.924, 95%CI: 0.737 to 1.112). A significant multiplicative allometric model including body mass, fat-free mass, CA, training experience and skeletal maturity status was also obtained (R = 0.684; R^2^ = 46.2%).

**Conclusion:**

Stature has limitations as a valid size descriptor of LVM. Body mass, fat-free mass, training experience, CA, body mass and skeletal maturity status were relevant factors contributing to inter-individual variability in LVM.

## Background

Growth refers to changes in body size, and adolescence is the interval of major changes in height, mass, proportions and composition [1]. The adolescent changes also influence the growth of specific organs, which in turn affects function. Cross-sectional [2] and longitudinal [3, 4] studies have reported that left ventricular mass (LVM) increases during childhood through adolescence. Because age-associated variation in cardiac dimensions is due, in part, to growth related increments in body size, LVM is routinely expressed relative to stature, mass or body surface area (BSA) [5–7]. Systematic training for specific sports during childhood and/or adolescence may influence left ventricular wall thickness (LVWT) and/or increments in the left ventricular cavity. These training adaptations may lead to challenges in diagnosing conditions such as dilated cardiomyopathy or hypertrophic cardiomyopathy [6].

The aforementioned structural and functional adaptive changes to the left ventricle have been labelled “athlete’s heart” [8] with LVM being the most common indicator of these cardiac adaptations [9, 10]. Chronic volume loads generally result in an increase in end-diastolic diameters and by inference in LVM; these consequently contribute to eccentric hypertrophy [11]. Intra- and inter-individual variability in cardiac variables in general and LVM in particular are associated with participation in sport but observed changes vary with type of sport [12]. It is also suggested that cardiac dimensions are associated with metabolically active tissues, mainly fat-free mass (FFM) [13–15]. Among 73 male roller hockey players 14–16 years of age, for example, estimated FFM was the best single predictor of inter-individual variance in LVM [16]; however, the results also suggested that biological maturity status should also be considered alongside stature (the traditional size descriptor) to index LVM. On the other hand, there is evidence that fat mass (FM) is also an independent and positive predictor of LVM in children and adolescents not engaged in youth sport [17, 18].

Historically, ratio standards have been frequently used to interpret physiological and morphological dimensions among individuals, including athletes, who vary in body size and composition. Stature (cm) and BSA (cm^2^) are, respectively, linear and bi-dimensional, while body mass and FFM are tri-dimensional variables. Allometric models have been suggested as an effective option for partitioning the effects of body size in order to derive a “size free” (dimensionless) expression of physiological parameters, e.g., maximum oxygen uptake in liters [19, 20] or LVM expressed in grams [6, 16, 21]. Since variation in body mass and composition is associated with growth, maturity status and also systematic training [1, 22, 23], proportional allometric models have been recommended among adult males and females [20]. Studies of youth athletes, particularly male hockey players, have addressed the independent and combined effects of variables such as chronological age (CA), maturity status (skeletal age, SA), and training experience with one or more body size descriptors (usually stature, body mass, FFM) on peak oxygen uptake [24] and LVM [16]. However, studies of LVM relative to body size have focused on adults and male adolescent athletes. Data are still lacking for female adolescent athletes.

The adolescent growth spurt differentially impacts attained stature and mass in youth of both sexes. Peak height velocity (PHV) occurs, on average, 2 years earlier in girls than in boys and tends to be less intense in girls [1]. Growth during the adolescent spurt has a marked impact on sex differences in body mass and composition. This is perhaps most marked in the linear increase in FFM among boys during adolescence, while the corresponding adolescent increase in FFM tends to reach a plateau in girls in association with a linear increase in adipose tissue. Therefore, it may be hypothesized that allometric models based on samples of male adolescent athletes may not be generalized to explain intra- and inter-individual variation in LVM among adolescent females. In this context, the objective of the present study was to examine the contribution of CA, skeletal maturity status, training experience and body size descriptors to inter-individual variability of LVM among adolescent female adolescent soccer players using an allometric modelling approach.

## Methods

### Procedures

The research was approved by the *Ethics Committee* of the *University of Coimbra* and a signed institutional agreement with the *Portuguese Institute of Sports*. Participants voluntarily visited the *Center for Sports Medicine* as part of the required medical examination for registration in the *Portuguese Soccer Federation* (Law 204/2006; act 11/2012). Parents or legal guardians and the players provided written consent; the players were informed that their participation was voluntary and that they could withdraw from the study at any time. During the visit to the medical Center, a radiograph of the left hand-wrist was taken for the purpose of SA estimation, echocardiography was conducted, and a series of anthropometric dimensions were measured. Each of the protocols was conducted by qualified Center personnel in the respective domains.

### Participants

The sample included 228 female soccer players 11.8–17.1 years (14.6 ± 1.1 years). All players were registered in competitive clubs affiliated with the *Portuguese Soccer Federation*. Inclusion criteria were engagement in formal training and competition for at least one complete year, Caucasian ethnicity, no symptoms of underlying cardiovascular disease, and no family history of cardiovascular-related mortality. Training experience was expressed as years of participation in competitive soccer at the club level, including registration with the *Portuguese Soccer Federation*. Individual information was obtained by interview on the day of observation and confirmed in consultation with institutional records of the Federation.

### Chronological age (CA) and maturity status

CA was calculated as the difference between date of the clinical examination and date of birth. SA was estimated with the Fels method [25], which includes maturity indicators for each of the 22 bones of hand-wrist and ratios of epiphyseal-diaphyseal widths. Grades and measurements for each indicator were entered into the Felshw 1.0 software (Felshw 1.0, Software Lifespan Health and Research Center, Departments of Community Health and Pediatrics, Booshoft School Medicine, Wright State University Dayton Ohio, USA) to derive an estimate of SA and the associated standard error. The same trained observer assessed all radiographs. The maturity status of each individual was subsequently classified [26] as late (SA younger than CA by more than 1.0 year), average or on time (the difference between SA and CA was within the band of − 1.0 years to + 1.0 years), early (SA older than CA by more than 1.0 years), or mature (SA is not assigned).

### Anthropometry

Body dimensions were measured following standardized procedures [27]. Stature was measured to the nearest 0.1 cm using a stadiometer (model 98.603, Holtain Limited Crosswell, Crymych, UK) and body mass was measured to the nearest 0.1 kg using a digital scale (SECA, model 770, Hanover, MD, USA). Skinfold thickness was measured to the nearest 0.5 mm at two sites, triceps and medial calf using a Lange caliper (Beta Technology Incorporated Cambridge, Maryland, USA). Body fat (fat mass, FM) as a percentage of body mass (%FM) was estimated from the two skinfolds using Eq. 1 recommended for female adolescents of White/European ancestry [28]. Absolute FM and FFM were derived.

1 $$ \%\mathrm{FM}=0.610\ \mathrm{x}\ \left(\mathrm{triceps}\ \mathrm{skinfold}+\mathrm{medial}\ \mathrm{calf}\ \mathrm{skinfold}\right)+5.1 $$

### Echocardiography

Resting echocardiographs were taken with a Vivid 3 ultrasound machine with a 1.5 to 3.6 MHz transducer (GE Vingmed Ultrasound, Horten, Norway). Two-dimensional images (recorded at 100 mm/s) were used to derive M-mode echocardiograms for direct visualization. Measurements of the internal dimension of the left ventricle at end diastole (LVIDd), septal wall thickness at end diastole (SWTd), and posterior wall thickness at end diastole (PWTd) were made following the procedures of the American Society of Echocardiography. Intra-observer technical errors of measurement and variability based on echocardiograms of 20 randomly selected adolescents measured twice within a one-week interval were previously reported [29]. Technical errors and 95% confidence levels were: LVIDd, 0.17 mm (95% LOA, 1.95–2.28 mm, %CV = 0.3, 95% LOA: 4.1–4.8%); SWTd, 0.02 mm (95% LOA, 0.30–0.34 mm, %CV = 0.3, 95% LOA, 4.2–4.8%); and PWTd, 0.06 mm (95% LOA, 0.45–0.56 mm, %CV = 0.8, 95% LOA, 6.5–8.1%). LVM was estimated using Eq. 2 [30] and relative wall thickness (RWT) was calculated using Eq. 3 [31]:

2 $$ \mathrm{LVM}=0.8\times \left\{\right(1.04\left[\mathrm{LVIDd}+\mathrm{PWT}+\mathrm{SWTd}\Big){}^3-{\left(\mathrm{LVIDd}\right)}^3\right]\Big\}+0.6 $$

3 $$ \mathrm{RWT}=\left(2\times \mathrm{PWTd}\right)/\mathrm{LVIDd} $$

### Analyses

Descriptive statistics were calculated and normality of distributions checked. Pearson correlations were used to estimate relationships among CA, SA and training experience in years, on one hand, and body size descriptors (stature, body mass and FFM) and echocardiographic parameters, on the other hand. Pearson correlations were also used to examine associations between the body size descriptors and parameters of LVM (simple and derived variables). Magnitude of the correlation coefficients was interpreted as follows [32]: trivial (r < 0.10), small (0.10 ≤ r < 0.30), moderate (0.30 ≤ r < 0.50), large (0.50 ≤ r < 0.70), very large (0.70 ≤ r < 0.90) and nearly perfect (r ≥ 0.90). Simple allometric models following procedures proposed by Nevill, Ramsbottom and Williams [19] and Nevill and Holder [20] were subsequently applied to the total sample:

4 $$ y=a.{x}^k.\varepsilon $$

5 $$ \text{In}\, y = \text{In}\, a + k .\text{In}\, x + \text{In}\, \varepsilon$$

Equation 5 corresponds to the natural logarithmic transformation of Eq. 4. It permitted the determination of the constant and power function for each size descriptor. In both equations, *y* corresponded to LVM, while *a* and *k* were, respectively, the constant and scaling exponents. Simple allometric models were validated by the inspection of the correlations between scaled LVM and the respective independent variables (size descriptors). The influence of size descriptors was removed when the coefficients of correlation approached zero. Finally, multiplicative allometric models were derived by combining size descriptors (stature, body mass, FFM), CA, years of training and skeletal maturity status (coded as dummy variables; the 65 skeletally mature participants were not considered in the simple and multiplicative allometric models). Backward stepwise multiple regression with *p* < 0.10 as the criteria for removal was used to develop a parsimonious model. This procedure reduces collinearity among independent variables. Diagnostic statistics to evaluate the proportion of variability in an independent variable that was not explained by the other independent variables (tolerance) were used to examine multicollinearity for the final models. The variance inflation factor (VIF) was also calculated. Variables were retained if tolerance was ≥0.1 and VIF was > 10 (to an R^2^ of 0.90). For each allometric model, the coefficient of determination (R^2^) was calculated to estimate the explained variance.

6 $$ \ln\ \left(\mathrm{LVM}\right)={k}_1.\ln\ \left(\mathrm{stature}\ \mathrm{in}\ \mathrm{cm}\right)+{k}_2.\ln\ \left(\mathrm{body}\ \mathrm{mass}\ \mathrm{in}\ \mathrm{kg}\right)+{k}_3.\ln\ \left(\mathrm{FFM}\ \mathrm{in}\ \mathrm{kg}\right)+a+{b}_1.\left(\mathrm{CA}\ \mathrm{in}\ \mathrm{years}\right)+{b}_2.\left(\mathrm{training}\ \mathrm{experience}\ \mathrm{in}\ \mathrm{years}\right)+{b}_3.\left(\mathrm{maturity}\ \mathrm{status}: late\  vs\  average; late\  vs\  early\ maturing,\mathrm{with}\ \mathrm{late}\ \mathrm{maturing}\ \mathrm{being}\ \mathrm{zero}\right)+\ln\ \upvarepsilon $$

Differences between skeletal maturity groups in size descriptors (stature, body mass, FFM) and in absolute and scaled values of LVM were graphically compared. The magnitude of mean differences between maturity groups was interpreted using Cohen’s d value as follows [32]: < 0.20 (trivial), 0.20–0.59 (small), 0.60–1.19 (moderate), 1.20–1.99 (large), 2.00–3.99 (very large), > 4.00 nearly perfect.

Statistical analyses were done with SPSS version 20.0 (SPSS Inc., IBM Company, N.Y., USA) and Graphpad Prism (version 5.00 for Windows, GraphPad Software, San Diego California USA, www.graphpad.com). Alpha level was set at 0.05.

## Results

Descriptive statistics for training experience, CA, SA, stature, body mass, BSA, body composition and echocardiographic parameters are summarized in Table 1. The distribution of players by maturity status (SA minus CA) was also indicated. CA was significantly correlated with stature (r = 0.19, *p* < 0.05), BSA (r = 0.21, *p* < 0.01), body mass (r = 0.18, *p* < 0.05), FFM (r = 0.21, *p* < 0.01) and LVM (r = 0.13, *p* < 0.05), but the correlations were low. Mean SA was advanced, on average, by approximately 0.65 year, relative to mean CA. SA was moderately correlated with FFM (r = 0.39, *p* < 0.01), BSA (r = 0.41, *p* < 0.01) and body mass (r = 0.41, *p* < 0.01). Correlations between training experience and several variables were lower but significant: negative with %FM (r = − 0.15, *p* < 0.05); positive for cardiac variables: ISWTd (r = 0.28, *p* < 0.01), PWTd (r = 0.25, *p* < 0.01), LVM (r = 0.22, *p* < 0.01) and the LVM index (r = 0.29, *p* < 0.01). Means for LVIDd, ISWTd and PWTd were 43.5 mm, 7.6 mm and 7.5 mm, respectively, in the skeletally mature players.

**Table 1:** Descriptive statistics and correlations between chronovariables, size and echocardiograph parameters (*n* = 228)

Variable	unit	descriptive statistics					normality (Kolmogorov-Smirnov)		correlations					
		range (min; max)	mean		standard deviation	f			CA		SA		training experience	
			value	(95% CI)			value	*p*	r	p	r	p	r	p
Training experience	years	(2; 9)	5.3	(5.0 to 5.5)	2.1		0.149	< 0.01						
Chronological age	years	(11.84; 17.05)	14.63	(14.49 to 14.77)	1.11		0.076	< 0.01						
Skeletal age^a^	years	(11.46; 17.92)	15.28	(15.04 to 15.53)	1.54		0.067	0.07						
Skeletal maturity:														
delayed	f					25								
average	f					51								
advanced	f					87								
mature	f					65								
Stature	cm	(136.0; 182.2)	161.3	(160.4 to 162.2)	6.8		0.056	0.08	0.192	< 0.05	0.241	< 0.01		
Body surface area	m^2^	(1.07; 2.05)	1.59	(1.57 to 1.61)	0.2		0.057	0.07	0.209	< 0.01	0.413	< 0.01		
Body mass	kg	(29.5; 101.0)	56.7	(55.2 to 58.1)	11.0		0.090	< 0.01	0.176	< 0.01	0.405	< 0.01		
Fat mass	%	(7.5; 51.6)	25.4	(24.4 to 26.4)	7.7		0.075	< 0.01			0.230	< 0.01	−0.148	< 0.05
	kg	(3.3; 51.4)	15.0	(14.0 to 16.0)	7.5		0.165	< 0.01			0.292	< 0.01		
Fat-free mass	kg	(26.2; 62.5)	41.7	(40.9 to 42.5)	5.8		0.051	0.20	0.213	< 0.01	0.391	< 0.01		
LVIDd	mm	(28.9; 56.1)	45.2	(44.7 to 45.7)	3.6		0.077	< 0.01			0.242	< 0.01		
ISWTd	mm	(5.4; 10.2)	7.7	(7.6 to 7.8)	0.9		0.064	0.03			0.203	< 0.01	0.288	< 0.01
PWTd	mm	(5.0; 9.6)	7.5	(7.4 to 7.6)	0.8		0.097	< 0.01					0.249	< 0.01
LVM	g	(50; 185)	107	(104 to 110)	22		0.049	0.20	0.131	< 0.05	0.274	< 0.01	0.222	< 0.01
LVM index	g.m^−2^	(33; 107)	67	(66 to 69)	11		0.023	0.20					0.286	< 0.01

*CA* Chronological age, *SA* Skeletal age, *f* frequency, *LVIDd* Left ventricular internal dimension at end of the diastole, *ISWTd* Interventricular septal wall thickness at end of the diastole, *PWTd* Posterior wall thickness at end of the diastole, *LVM* Left ventricular mass, *LV index* Left ventricular mass index = LVM / body surface area, *f* Absolute frequency, 95% CI (95% confidence intervals)

^a^*n* = 163; players classified as skeletally mature (*n* = 65) were not considered in the analysis

**Fig. 1:**
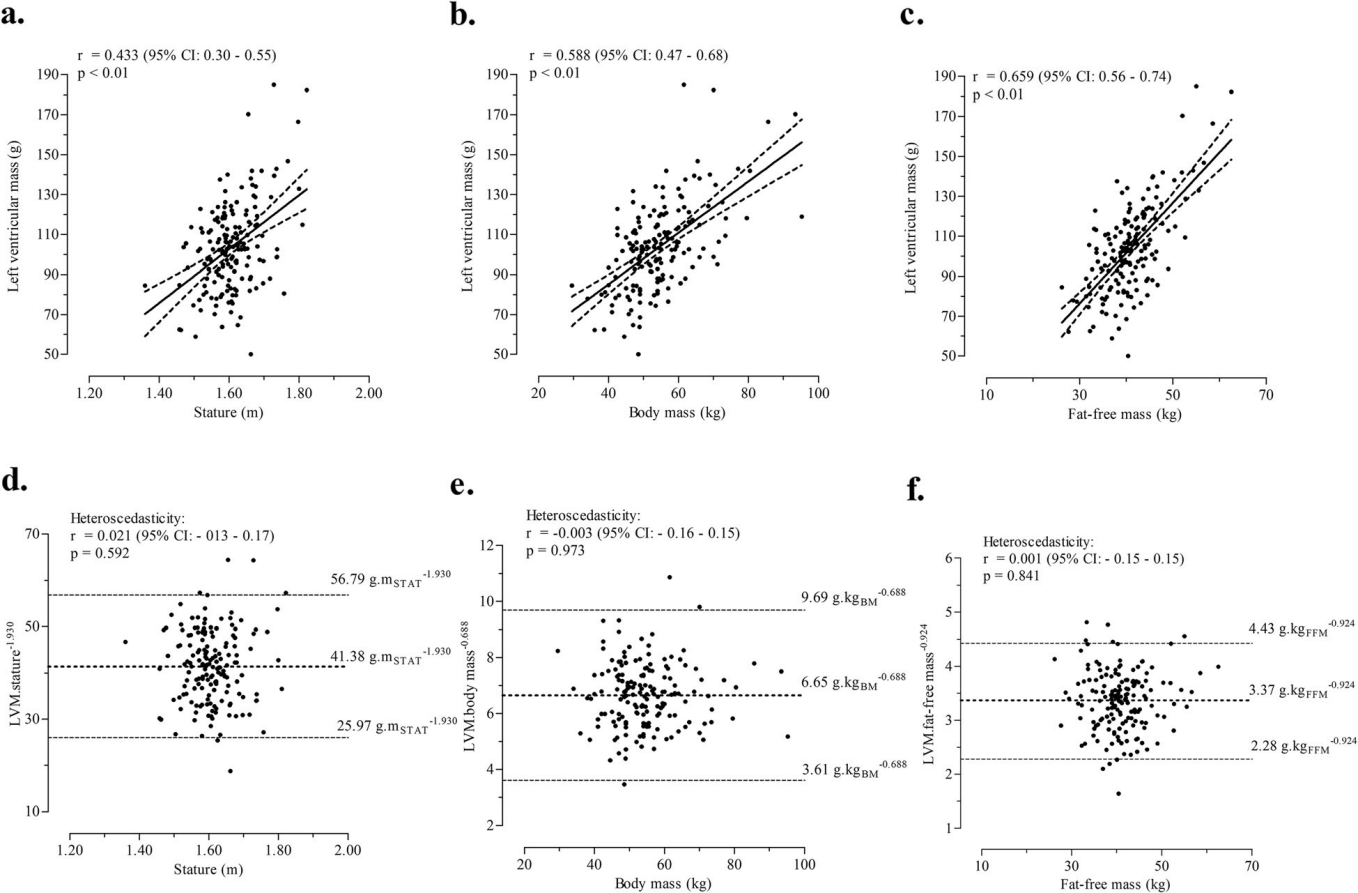
Relationship of LVM to stature (**a**), body mass (**b**) and fat-free mass (**c**), and correlations between power functions and respective size descriptors (**d**, **e** and **f**)

Correlations between size descriptors and dependent variables varied from moderate to large (Fig. 1a-c). Accordingly, simple allometric models between logarithmic transformations were calculated using stature, body mass and FFM as size descriptors to obtain dimensionless models aimed to explain inter-individual variability of LVM (Table 2). The allometric coefficients explained 16 to 37% of variance in LVM showing a linear relationship between LVM and FFM (k = 0.924, 95%CI: 0.737 to 1.112). Power function exponents for stature (*k* = 1.930, 95%CI: 1.240 to 2.620) and body mass (*k* = 0.688, 95%CI: 0.536 to 0.840) were, respectively, above and below, the unit corresponding to linearity. Finally, correlations between scaled variables and LVM were negligible for all size descriptors, suggesting that the simple allometric models were effective to evaluate LVM independent of body size (Fig. 1d-f).

**Table 2:** Bivariate correlations and simple allometric models between LVM and size descriptors (*n* = 163)

X_i_: size descriptors	correlations between LVM and size X_i_			simple alometric models [ln (LVM) = ln (a) + *k*_*i*_ × ln (X_i_) + log (ε)]						correlation (X_i_, LVM/X_i_^k^)
	r	95% CI	(qualitative)	a	*k*_*i*_		model summary			
					value	(95% CI)	R	*R*^2^	*p*	
Stature	0.433	(0.299 to 0.550)	(moderate)	−5.185	1.930	(1.240 to 2.620)	0.399	0.159	< 0.01	0.021
Body mass	0.588	(0.477 to 0.680)	(large)	1.878	0.688	(0.536 to 0.840)	0.576	0.332	< 0.01	−0.003
Fat-free mass	0.659	(0.562 to 0.738)	(large)	1.199	0.924	(0.737 to 1.112)	0.608	0.366	< 0.01	0.001

*LVM* Left ventricular mass, *r* correlation coefficient, *95%CI* 95% confidence intervals, *k*_*i*_ scaling coefficient, ε error, *a* constant, *R*^2^ Explained variance

**Fig. 2:**
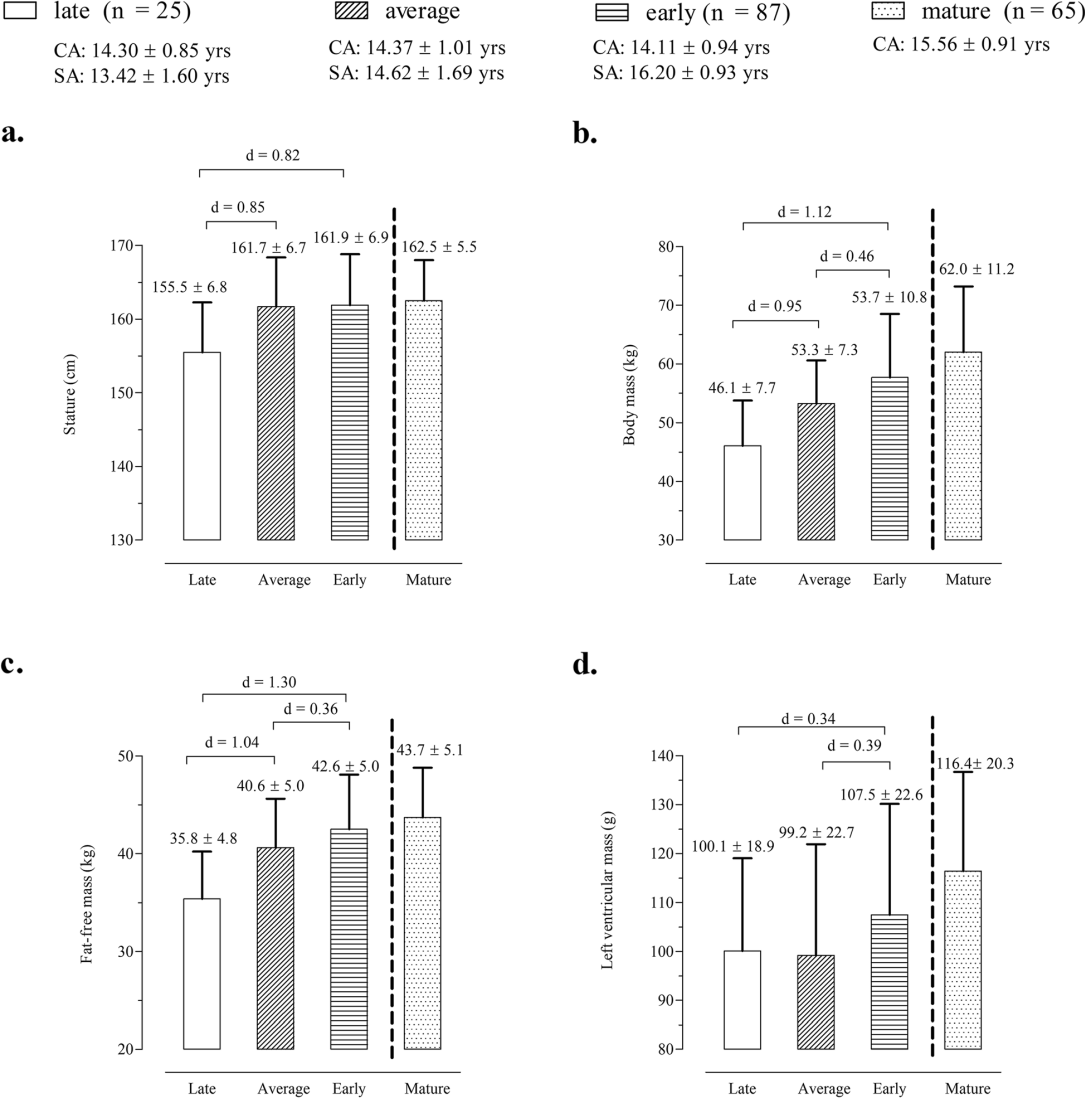
Mean values for stature (**a**), body mass (**b**), fat-free mass (**c**) and left ventricular mass (**d**) for the total sample of players by skeletal maturity status

The panels of Fig. 2 illustrate maturity-associated variation in stature, body mass, FFM and LVM. LVM showed the same maturity gradient (i.e. late < average < early) as noted for size descriptors. Comparisons of late and average maturing groups indicated consistently moderate Cohen’s d values (0.85 < d < 1.04). Corresponding comparisons between late and early maturing indicated magnitude differences ranging from moderate (d = 0.82 for stature; d = 1.12 for body mass) to large (d = 1.30 for FFM). Finally, differences between average and early maturing players tended to be trivial (stature: d < 0.20) or small (body mass: d = 0.46; FFM: d = 0.36).

Taking into account interrelationships among size descriptors and skeletal maturity status, it was decided to examine their multiplicative effects on heart size. Table 3 summarizes the results of multiplicative allometric modelling combining size descriptors, CA and training experience with maturity status as a dummy variable. The explained variance for LVM increased to 46%; the resulting equation was as follows:

**Table 3:** Multiplicative allometric modelling* of LVM combining size, CA, skeletal maturation and training (n = 163)

Predictors	constant	coefficients	*p*	collinearity		model summary ^a^			
				tolerance	VIF	R	R^2^ adjusted	F	p
						0.694	0.462	24.146	< 0.01
	1.070		< 0.01						
ln (body mass)		0.412	< 0.01	0.307	3.259				
ln (fat-free mass)		0.621	< 0.01	0.288	3.470				
Chronological age		−0.028	0.05	0.845	1.184				
Training experience		0.022	< 0.01	0.777	1.287				
Skeletal maturity status									
Late vs average		−0.137	< 0.01	0.397	2.520				
Late vs early		−0.116	< 0.01	0.339	2.952				

*VIF* Variance inflation factor, *R*^*2*^ Explained variance

^a^ln (LVM) = *k*_1_ × ln (stature) + *k*_2_ × ln (body mass) + *k*_3_ × ln (fat-free mass) + a + *b*_1_ × (CA) + *b*_2_ × (training years) + *b*_3_ × (maturity status: late vs average; late vs early maturing, with late maturing being zero) + ln ε

7 $$ \ln\ \left(\mathrm{LVM}\right)=1.070+0.412\times \ln\ \left(\mathrm{body}\ \mathrm{mass}\right)+0.621\times \ln\ \left(\mathrm{FFM}\right)-0.028\times \left(\mathrm{CA}\right)+0.022\times \left(\mathrm{training}\ \mathrm{experience}\right)+0\ \left(\mathrm{if}\ \mathrm{maturity}\ \mathrm{status}=\mathrm{late}\right),-0.137\ \left(\mathrm{if}\ \mathrm{maturity}\ \mathrm{status}=\mathrm{average}\right),\mathrm{and}-0.116\ \left(\mathrm{if}\ \mathrm{maturity}\ \mathrm{status}=\mathrm{early}\right). $$

**Fig. 3:**
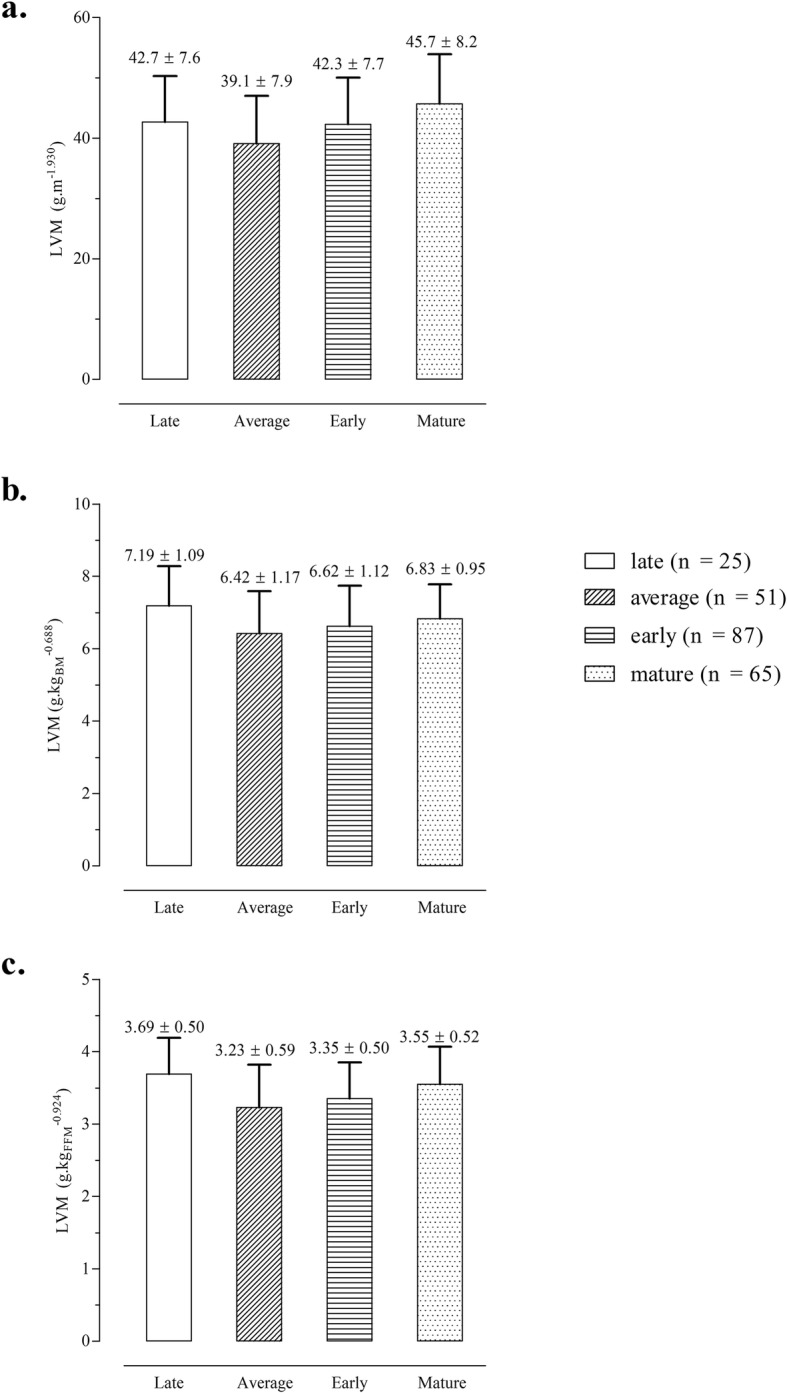
Means and standard deviations by skeletal maturity groups for scaled LVM expressed per unit of stature (**a**), body mass (**b**) and fat-free mass (**c**)

When using scaled LVM values, the maturity-associated gradient was attenuated, and adolescent female soccer players classified as delayed (late maturing group) showed similar values compared to other maturity groups (Fig. 3).

## Discussion

The contributions of CA, skeletal maturity status, training experience in competitive soccer and indicators of body size to inter-individual variability in LVM was considered among Portuguese adolescent female soccer players 11.8–17.1 years of age. The predicted variable (i.e., LVM) was interpreted as tri-dimensional and, not surprisingly, the contribution of the stature (uni-dimensional size descriptor) to the explained variance in LVM was relatively low (about 16%). Stature did not consistently enter the final multiplicative allometric model. On the other hand, FFM (tri-dimensional indicator) was the best single predictor of LVM, explaining 37% of the variance. Its scaling coefficient, *k* = 0.924 (95%CI: 0.737 to 1.112), suggested a linear relationship with LVM (geometric similarity). Body mass, another tri-dimensional indicator, had a scaling coefficient, *k* = 0.688 (95%CI: 0.536 to 0.840), that departed from linearity and suggested an elastic relationship between body mass and LVM. The final multiplicative allometric model suggested that body mass, FFM and training experience in soccer were directly associated with LVM, and after controlling for the preceding, average and early maturing players had a proportionally smaller LVM compared to late maturing peers (reference group in the analysis).

The adolescent female soccer players had a mean stature at the 50th percentile of the US reference data for girls of the same age [33], but a mean body mass between the 50th and 75th percentiles of the reference. The tendency for greater mass-for-stature may reflect their advanced skeletal maturity status, consistent with cross-sectional observations for Portuguese adolescent male soccer players [34–37]. The body mass index (BMI) each individual participant was also plotted relative to US age-specificz-scores [33] and the majority of female soccer players (*n* = 194) ranged between − 1.0 and + 1.0, while 26 players had BMIs that exceeded + 1.0. In addition, 103 of the soccer players were characterized by an excessive amount of fatness predicted from two skinfolds (> 25%), and the data showed a maturity-related gradient in %FM: early>average > late. It is thus possible that excess body mass-for-stature may reflect increased FM. Nevertheless, future studies should consider alternative assessments of body composition such dual-energyx-ray absorptiometry (DEXA) or air displacement plethysmography, may provide more accurate estimates of FM and FFM.

Absolute values for LVM, LVIDd and PWTd in the current study were comparable to those reported for 32 American female soccer players 13–18 years [38]. Interpretation and comparison of cardiac indicators across samples are influenced by body size and composition, but detailed information on the body dimensions of the American sample was not reported. Additionally, a slight increase in LV cavity and lower PWT were noted in the present sample of soccer players compared to female swimmers of the same age and similar average body masses [39]. It is possible that the results suggest eccentric remodelling independent of physiological adaptations to the haemodynamic loading associated with soccer participation [13, 31, 40, 41].

Theoretical allometric coefficients of *k* = 2.13 and *k* = 2.65 for stature have been adopted to normalize the effects of body size in LVM [6, 7, 14, 31, 42]. The simple allometric models in the current study, however, noted a lower exponent for stature (*k* = 1.930). The differences may reflect sampling variation, methodological constraints, statistical procedures, sex and/or age-associated variation. Moreover, stature only explained ≈16% of the variance in LVM and was not included in the proportional allometric model. Overall, the findings suggested that stature alone should not be considered to normalize or predict LVM. Similar results were also noted in 464 highly trained junior male and female athletes 14–18 years participating in cycling, soccer (males only), rowing, swimming and tennis, with small numbers in other sports [5].

It is possible that other size descriptors may be needed to normalize LVM. More recently, FFM based on DEXA was noted as the best size descriptor to compare LVM in 75 young adult females in static or dynamic sport activities [15]. Among dependent variables in the current study, FFM was the best explanatory predictor of LVM, confirming the influence of metabolically active tissues on cardiac output. Results of the present study also suggested a linear relationship between the logarithmic transformations of LVM and FFM. The utility of simple ratios to estimate cardiac output of LVM per unit of FFM was noted in studies of trained [13] and untrained adults [43] consistent with the theoretical range of geometric similarity, i.e., LVM is represented as a cubic expression and as such requires a 3-dimenisonal variable for normalization. On the other hand, the interpretation of LVM considering only FFM is limited by a lack of comparative studies in youth female sport participants.

Multiplicative allometric models are physiologically plausible and accommodate heteroscedasticity in the distribution of a variable, and thus provide a better statistical fit than simple models [44]. Not surprisingly, FFM combined with years of training in soccer and biological maturity status provided a better understanding of LVM than simple allometric models. The results were consistent with previous cross-sectional studies of adolescent sport [16] and non-sport participants [45] in showing that the interrelationships between growth and maturation are determinants of LVM. However, the contribution of SA per se was not a significant predictor of LVM in Portuguese male roller hockey players 14.5–16.5 years of age [16]. In contrast, results of the multiple backward regression analysis among adolescent female soccer players indicated that maturity status defined by SA minus CA was a significant determinant of LVM. SA provided perhaps the most accurate estimate of maturity status, i.e., the state of maturation of the hand-wrist bones at the time of observation [1]. By inference, SA should be expressed relative to CA for inclusion in multiplicative allometric models.

A gradient of maturity associated differences in size and LVM were noted in the soccer players (early > on time > late maturing girls). Early maturing players tended to be heavier and relatively fatter and presented a larger LVM compared to average and late maturing players (Fig. 2). This was consistent with observations for 6029 Flemish girls 6–16 years of age which showed a positive relationship between fatness and SA based on the Tanner–Whitehouse 2 method [46]. The trends thus suggested that absolute values of LVM were significantly influenced by early maturation which in turn was related to body composition, specifically pubertal gains in FM. Although FM does not have a strong relationship with LVM, sports participation was associated with changes in FM and FFM [22] which may be a potential explanation for the inclusion of years of training in soccer and FFM in the final allometric model. Nevertheless, the multiplicative allometric model indicated that differences in LVM among maturity groups were reversed when body mass, FFM, CA and training experience were appropriately controlled. After controlling for body size descriptors (i.e., scaled LVM output), there were no substantial differences amongst female adolescent soccer players contrasting in maturity status (Cohen’s d values were less than 0.20 as showed in Fig. 3a-c). A study of peak oxygen uptake among 54 adolescent females (10.7–13.5 years) to evaluate allometric models for concurrent size descriptors (stature, body mass and FFM) noted that scaled performance did not differ according to categories of self-assessed pubic hair development [47]. Among 59 male adolescent basketball players, those aged 14 years and classified in stage 3 for clinically assessed pubic hair development (mid-puberty) performed, on average, better on the 20-m shuttle run test than peers of the same age classified in stage 5 (post-pubertal) [48]. Overall, the available studies show that early maturing adolescents tend to be taller, heavier and stronger, but may not demonstrate superior performance in aerobic fitness tests.

Generally comparable results showing an influence of predicted maturity status based on predicted maturity offset, i.e., time before or after PHV on absolute values of peak force were noted in a cross-sectional study of 157 female soccer players combined across four competitive age groups, U10 through U16 [49]. However, conclusions based on predicted maturity offset as an indicator of maturity status across this broad age range should be interpreted with caution given limitations of the equation used to predict maturity offset in girls. More specifically, predicted offset and in turn predicted age at PHV are affected by CA at prediction and has major limitations in early and late maturing girls defined by observed ages at PHV in two validation studies based on longitudinal samples [50, 51].

The distinction between physiologic increases in LVWT in athletes (i.e., athlete’s heart) and hypertrophic cardiomyopathy accounts for about one-third of all exercise-related sudden cardiac deaths in trained athletes aged < 35 years old [52–54], and intense competitive sport is not recommended [55]. To define physiologic limits of left ventricular hypertrophy in elite adolescent athletes, echocardiography was performed among 720 elite adolescent athletes (75% male) aged 14–18 years participating in ball, racket, and endurance sports, and in 250 healthy sedentary controls of similar age, sex, and body surface area [56]. Only a small proportion of athletes exhibited a LVWT exceeding upper limits and authors concluded that compared with controls, adolescent athletes had greater absolute LVWT. However, it should be noted that many sports tend to recruit/select and promote young athletes that have larger body sizes [23, 35, 48] and interpretation of both wall thickness and cavity diameter should be done according to principles of geometric similarity of heart size to body size [19]. LVM is a tri-dimensional variable and, consequently, it is not expected to have a linear relationship with stature in cm. Linearity is, however, expected between LVM and FFM which is the metabolically active component of body mass. Athletes exposed to systematic training tend to be characterized by a larger FFM [1, 22]. Identification of athletes exceeding physiological limits is thus recommended. In addition, skeletal maturity status is an additional source of inter-individual variation in LVM, but does not correspond to any abnormality when LVM is scaled properly.

Although the present study considers a previously under studied population (adolescent female soccer players) and includes of a valid and established indicator of maturity status, specifically SA, several limitations of the present study should be noted. The sex-specific equation for predicting %FM from two-skinfold thicknesses has a standard error of estimate of 3.8% [28]. FM was estimated as predicted %FM × body mass, and FMM was derived by subtraction (body mass - FM = FFM). Based on the two skinfolds used in the present study, %FM was 18.6 ± 7.2% in a combined sample 126 youth soccer players (mean age: 13.3 years, 86 boys, 40 girls) and was lower than estimated %FM based on DEXA, 21.9 ± 5.8% [57]. Unfortunately, the prediction equations are different for boys and girls so that comparisons with the combined sample should be interpreted with caution. The equation for boys [28] was also used in adolescent roller hockey players [16]; estimates of FM and FFM derived from predicted %FM were significant contributors to inter-individual variability in LVM using allometry (FM: r = 0.56, 31% explained variance; FFM: r = 0.51, 26% explained variance).

Future research is needed to examine intra- and inter-individual variability in LVM associated with specific aspects of sport training and participation and internal and external markers of training load such as minutes and sessions, and ratings of perceived exertion. Moreover, characteristics of training process are generally specific for initiates, juveniles or juniors (competitive age groups by the Portuguese Soccer Federation). Although the sample size in the present study (*n* = 163) was larger than in previous studies, the cross-sectional design does not support a cause-effect relationship between size descriptors and cardiac remodeling. Finally, although echocardiography is still the most widely used method for assessing LVM, cardiac magnetic resonance imaging is considered the gold standard for determining LVM.

## Conclusions

Results of this cross-sectional study of adolescent female soccer players indicated that inter-individual variance in LVM is, in part, explained by skeletal maturity status which affects body size and composition. Specifically, a larger body size tended to be associated with early maturing participants. Skeletal maturation, training experience, body size and composition should be considered in the interpretation of an athlete’s heart. The study also highlighted the utility of multiplicative allometric models for understanding LVM among adolescent girls participating in competitive soccer. Interpretation of echocardiography data from adolescent athletes apparently exceeding the physiologic limits of left ventricular size may require the assessment of body composition and SA.

## Data Availability

The database supporting the conclusions of this article is available from the corresponding author on reasonable request.

## Abbreviations

LVM: Left ventricular mass
BSA: Body surface area
FFM: Fat-free mass
FM: Fat mass
%FM: Percentage of fat mass
CA: Chronological age
SA: Skeletal age
PHV: Peak height velocity
LVIDd: Internal diameter of left ventricle at end diastole
SWTd: Septal wall thickness at end diastole
PWTd: Posterior wall thickness at end diastole
LVWT: Left ventricular wall thickness
RWT: Relative wall thickness
DEXA: Dual-energy x-ray absorptiometry
BMI: Body mass index
